# Ancient Living Organisms Escaping from, or Imprisoned in, the Vents?

**DOI:** 10.3390/life7030036

**Published:** 2017-09-15

**Authors:** J. Baz Jackson

**Affiliations:** School of Biosciences, University of Birmingham, Edgbaston, Birmingham B15 2TT, UK; j.b.jackson@bham.ac.uk

**Keywords:** natural pH gradient, hydrothermal vents, chemiosmotic theory, origin of life

## Abstract

We have recently criticised the natural pH gradient hypothesis which purports to explain how the difference in pH between fluid issuing from ancient alkali vents and the more acidic Hadean ocean could have driven molecular machines that catalyse reactions that are useful in prebiotic and autotrophic chemistry. In this article, we temporarily suspend our earlier criticism while we consider difficulties for primitive organisms to have managed their energy supply and to have left the vents and become free-living. We point out that it may have been impossible for organisms to have acquired membrane-located proton (or sodium ion) pumps to replace the natural pH gradient, and independently to have driven essential molecular machines such as the ATP synthase. The volumes of the ocean and of the vent fluids were too large for a membrane-located pump to have generated a significant ion concentration gradient. Our arguments apply to three of the four concurrent models employed by the proponents of the natural pH gradient hypothesis. A fourth model is exempt from these arguments but has other intrinsic difficulties that we briefly consider. We conclude that ancient organisms utilising a natural pH gradient would have been imprisoned in the vents, unable to escape and become free-living.

## 1. Introduction

It is widely understood that energy sources from the environment were utilised to drive primitive metabolism and polymerisation reactions at the origin of life. Russell et al. [1] proposed an ingenious hypothesis in which a natural pH gradient (ΔpH) was formed across an inorganic membrane, somehow interposed between alkaline fluid issuing from submarine hydrothermal vents and the more acidic Hadean ocean. Inorganic molecular machines embedded in the inorganic membrane were thought to have used the energy released from a current of protons flowing down the natural ΔpH to drive useful chemistry such as pyrophosphate synthesis and CO_2_ reduction. The hypothesis was inspired by the chemiosmotic theory advanced by P Mitchell in the 1960s and now recognised as providing a most important basis for energy coupling reactions in modern organisms [2,3,4]. The natural pH gradient hypothesis has been further developed in numerous publications from the research groups of MJ Russell, W Martin, N Lane, and their colleagues, whom we shall collectively refer to as the RML groups. It is pivotal to the wider views of the RML groups on their perception of autotrophy at the origin of life. The basis for the natural pH gradient hypothesis was recently criticised [5]. Flaws in the physicochemical descriptions were revealed, and inefficiencies that would result from driving nanometre-sized molecular machines using electrochemical ion gradients across micrometre thick membranes were highlighted. In a subsequent paper, claimed experimental support for the hypothesis [6] was challenged [7]; see also [8]. The reduction of CO_2_ by H_2_ to formate in an “origin of life reactor” [6] was shown by a simple calculation not to be driven by a natural ΔpH, but to be a simple consequence of that reaction approaching equilibrium in the absence of an external energy source. 

The RML groups [9,10] maintain that, as well as having a crucial role at the origin of life, natural pH gradients continued to drive molecular machines in primitive living organisms in the vents until the time of the last universal common ancestor (LUCA). In this article, we shall temporarily set aside objections to the hypothesis, as it applies to the *origin* of life [5,7], while we consider problems associated with the LUCA “escaping from the vents”, relinquishing the natural ΔpH, and adopting a modern energy conservation system to become a free-living organism. For purposes of discussion we will provisionally accept the RML definition of the LUCA, though it should be appreciated that this definition is contentious [11].

## 2. Escaping from the Vents by LUCA Bleb Membranes

**Figure 1:**
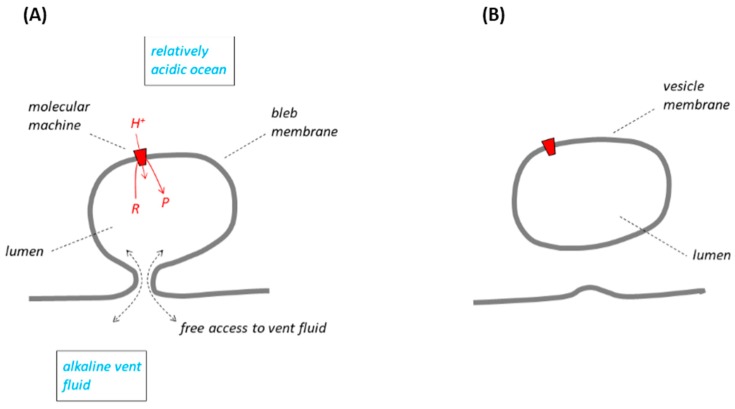
The “bleb” model of membranes escaping from the vents. The figure is of the last universal common ancestor (LUCA) (following the definition of [10]) with a *lipid* bleb membrane (see text) in grey, and according to the description of structures in [9,12,13,14]; the lipid has replaced earlier *inorganic* membranes. *R* and *P* are the reactants and products of a protein, natural pH gradient (ΔpH)-utilising molecular machine (red trapezoid). It is emphasised that the term “ΔpH-utilising molecular machine” is intended throughout the present work as broad description of any device, inorganic or protein, that uses the energy of a pH gradient, natural or otherwise, to drive useful chemistry by whatever mechanism. In part (**A**), the machine uses the proposed natural ΔpH between the alkali solution of the vent and the more acidic Hadean ocean to drive conversion of *R* to *P*. In part (**B**), the bleb has sealed off (or bubbled off) from its adjacent membrane and no longer has access to the natural ΔpH (see text).

The RML groups have several concurrent models for the inorganic membranes that might have separated alkaline fluid issuing from the ancient vents and the relatively acidic ocean water. In our opening paragraphs, we shall take the model shown in Figure 1A (from [9,12,13,14]) as a basis for discussion, although we shall call the membrane a “bleb” rather than a “vesicle” [9], “compartment” [13] or “pore” [14]. Subsequently, we shall establish that most (though not all) of the other membrane models from the RML groups reduce to the same principles as those in the bleb description. In the LUCA, the inorganic membrane, thought to have been in place at the origin of life, had been replaced by organic (lipid) membrane [10,15], but see [11]. The bleb is topologically and energetically indistinguishable from the “minimal membrane” that was shown in Figure 1 of [5]. Before escaping from the vents, the LUCA of Lane and Martin would have had genes, proteins and a small-molecule metabolism [10]; these must have been largely confined to the lumen of the bleb to ensure their function, and prevented from diffusing through the narrow basal constriction into the large volume of the vent where they would have been lost. Problematically, the basal constriction *at the same time* must have been wide enough to allow unfettered access of the alkaline vent fluid to the bleb lumen to permit replenishment of the natural pH gradient, the energy supply, but we shall not discuss this apparent contradiction any further. According to Lane and Martin [10] the LUCA would have had membrane-located molecular machines, but by this time evolved into proteins such as ATP synthase, which could utilise the energy of the natural pH gradient.

To escape from the vents, it was proposed [10,13,16] that the LUCA membrane “bubbled off” or “sealed off” from its associated vent membrane to allow the organism its independence. Figure 1 illustrates the principle. The LUCA would have become *vesicular*, entirely bounded by a single membrane and surrounded by ocean water. During the sealing-off process, the lumen of the LUCA membrane system would have been completely disconnected from the supply of alkaline vent fluid, necessary to maintain the natural ΔpH. The ΔpH-dependent molecular machines in the membrane would therefore have continued only briefly to operate with the residual, trapped alkaline solution originating from the vent. The pH gradient across the vesicular membrane would soon have been either consumed by the molecular machines or dissipated by proton and/or hydroxide ion leakage. Had ΔpH not been restored, this would have quickly resulted in failure of the molecular machines, and death of the organism. A way to avoid this catastrophe, considered [9,10], was that the LUCA membrane acquired a protein *proton pump* in the period *before* the sealing-off process began. This proton pump would have had an energy source, perhaps a set of inorganic redox reactions, and catalysed proton translocation from the *bleb* lumen (at that time still contiguous with the vent) into the ocean. After sealing off, it would have continued this activity but then, of course, pumping protons from the *vesicle* lumen into the ocean, the resulting pumped ΔpH now supplanting the former natural ΔpH and driving the molecular machines in a chemiosmotic circuit similar to that in modern organisms. At this point the LUCA would thus have been able to function independently of any natural ΔpH as it escaped from the vents.

However, there is a strong, perhaps fatal, argument against such an explanation: to be effective the proposed new proton pump must have emerged *in readiness* before the LUCA sealed off from the vent, but during that earlier period it would have had no value to the organism. It is re-emphasised that the coupling membrane of the natural pH gradient hypothesis (by the RML group definition) would have lain between two very large reservoirs with immense buffering capacities: the alkali vent and the Hadean ocean. An emerging proton pump located within this coupling membrane, and translocating H^+^ from the vent to the ocean before sealing off, would not itself have given rise to any significant ΔpH (we assume, and it is tacitly assumed in [10], that electric potential changes during this process are small: Δψ remained close to zero). Thus, the uptake of H^+^ from the vent and the release of H^+^ into the ocean by the pump would have had a negligible effect on the pH of these two, high-volume, aqueous phases. Compare this with the postulated, large, natural ΔpH created by powerful geochemical (serpentinization) activity beneath the vent and, according to [1,17,18], by massive CO_2_ dissolution into the ocean. Before sealing off, the ΔpH created across the LUCA membrane by a putative pump would thus have been *as nothing* compared with the natural pH gradient. Only after sealing off, as the pump began to translocate protons from the *small volume* of the vesicle (Figure 1B), could it have successfully produced a significant ΔpH. A set of oxidation-reduction reactions to drive the pump, and its synthesis under genetic control, might be imagined, but before sealing off there could have been no selective pressure to guide the development of the pump because the device would have had no function and would not have given any survival value to the organism. The advent of an evolved proton pump under these conditions was therefore highly unlikely.

Lane and Martin [10] put forward another suggestion for the management of energy conservation reactions in the LUCA membrane during the sealing-off process but, in my view, this would also have failed. They proposed that, before the sealing off began, the LUCA membrane acquired a protein Na^+^/H^+^ antiporter (designated SPAP), which coupled thermodynamically favourable H^+^ transport from the ocean to the vent with Na^+^ transport from the vent to the ocean: they argued that the SPAP would have used the energy of the natural pH gradient to build a Na^+^ concentration gradient across the membrane of the LUCA. Quite properly, it was recognised in [10] that the ATP synthase, and some other Δp-consuming protein molecular machines, in some modern organisms, are promiscuous: they can operate with either H^+^ or Na^+^. Lane and Martin [10] proposed that, before sealing off, the Na^+^ concentration gradient they expected to have developed across the membrane of the LUCA, as well as the natural pH gradient, could *both* have contributed to the driving force for the machines, using either Na^+^ or H^+^ as the translocated ion, for example, to make ATP. They supposed that under these conditions the ATP synthase would have become better adapted to Na^+^. An energy-dependent Na^+^ pump was thought then to have developed in the membrane (e.g., the Mtr protein in their Figure 2B) to contribute to the Na^+^ gradient, and therefore also to drive the molecular machine, and eventually to have taken over and powered ATP synthesis in a chemiosmotic Na^+^ circuit after sealing off. The argument behind this predicted turn of events was derived from an attempt to understand how/why proton-leaky early membranes later became proton-impermeable: it will not be reproduced here [10]. Suffice it to say that the activities of the postulated SPAP and Na^+^ pump would have been *unable* to generate a significant Na^+^ concentration gradient across the membrane of the LUCA before sealing off (Figure 1A). Thus, the translocation of Na^+^ from the very-large-volume vent into the very-large-volume ocean would *not* have produced significant changes in Na^+^ concentration in either of these two aqueous phases. The resulting, extremely small Na^+^ concentration gradient, whether from an SPAP or a Na^+^ pump, would have been quite insufficient, for example, to drive an ATP synthase before the sealing off process began. Once again, this revised hypothesis calls for the acquisition and evolution of proteins that had no survival value to the organism until later—until *after* the LUCA had sealed off from the vent. Perhaps with this in mind, it was argued [19] that the origin of an SPAP and a Na^+^ pump would have been more likely in “distal regions of the vent” where the release of alkaline fluid, and hence the generation of a natural ΔpH, were limited; see also [9]. This consideration does not help: before sealing off, a Na^+^-concentration gradient generated as a consequence of either SPAP or Na^+^ pump activity would have been energetically insignificant compared with the natural ΔpH *wherever* in the vent the membranes housing these proteins were located. No selective advantage from such a sodium gradient would arise from a good-for-nothing SPAP or pump activity before sealing off, and no evolutionary pressure to guide development of these proteins in the LUCA.

## 3. Escape from the Vents of Compartments from an Ocean-Floor Mound

**Figure 2:**
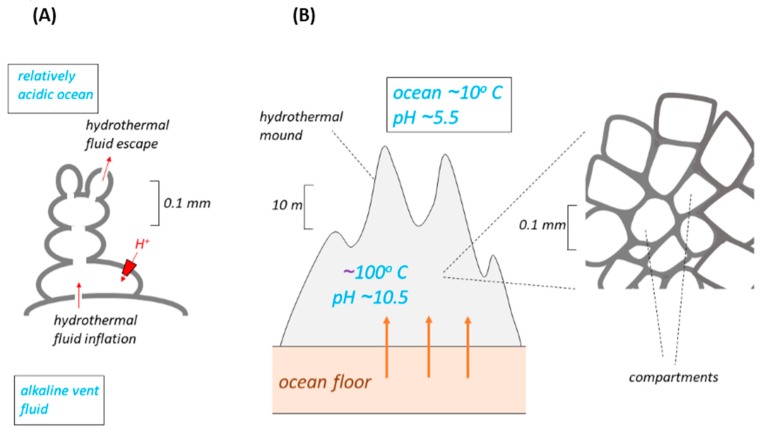
Hydrothermal mounds of inorganic membranes. *Inorganic* membranes are shown in dark grey. (**A**) is from [1]. The red trapezoid represents a ΔpH-utilising inorganic molecular machine. (**B**) The main part shows the dimensions of the proposed hydrothermal mound and is from [24,25,26]. The inset is of some of the billions [27] of compartments within the mound, from [19,20]. We assume the value printed across the mound is intended to represent the internal pH of the compartments, being fed by the alkaline vent fluids (bold orange arrows). At the same time, the interstitial spaces between compartments are thought to have been fed by percolating water from the relatively acidic ocean. A resulting large ΔpH across the inorganic membranes of each compartment would have driven molecular machines in those membranes (but see text). The caption for Figure 2 in [24] suggests that the thickness of the inorganic membranes was approximately 10 nm, but this is much less than that observed in laboratory-synthesised inorganic membranes [5,7]. Because ΔpH-utilising molecular machines may be located in membranes that are *shared* by two adjacent compartments (ostensibly a point not considered in [19,20]), their orientation and their direction of operation (forward/reverse) are difficult to define in this model (see Figure 2 in [5] and the accompanying comments); they are therefore not shown here.

Another model (Figure 2) of the membranes thought perhaps to have separated the alkaline vent fluid from the more acidic ocean water has either a mound or a column of 10–100 μm compartments gathered around ancient hydrothermal vents on the ocean floor [1,10,12,17,19,20,21,22,23,24,25,26]. The bioenergetics properties of this model (Figure 2) are essentially similar to those of the bleb. This is easy to see in the early versions of the model [1,17,21] where the number of compartments is small (Figure 2A). Clusters of interconnected iron monosulfide bubbles (botryoids) were inflated by hydrothermal alkaline solution at seepage sites. Their outsides were bathed in lower pH ocean water. The eventual escape of compartments from the clusters, following evolutionary replacement of the inorganic membranes by lipid, might thus have followed the mechanism proposed [10], but with all the attendant difficulties discussed above.

In later versions of this model [21,24,28] very large numbers of compartments, perhaps billions [27], form the mound, a “hatchery” several meters high. It is difficult to see how each compartment in such a mound could be fed individually and directly by alkali fluid from seepage sites, and how the interstitial spaces between the compartments could be *simultaneously* bathed by fresh ocean water, to maintain a ΔpH large enough to drive ΔpH-utilising molecular machines in the inorganic membranes. At best, we might expect a gradation of pH across the many compartments between vent and ocean, and consequently a greatly reduced ΔpH across each compartment membrane. However, notwithstanding this difficulty, the compartments in the mound still share the properties of a bleb. We must then conclude that the arguments developed above against the successful escape of the bleb-like compartments from a vent also apply to the compartments of a mound (whether large or small). Thus, the dissociation of any compartment from the mound would result in its demise, and this cannot be forestalled by the prior development of H^+^ pumps, SPAP proteins, or Na^+^ pumps [10] for similar reasons to those outlined above.

## 4. Escape from the Vents of Micropores (Microcompartments) in a Labyrinthine Network

**Figure 3:**
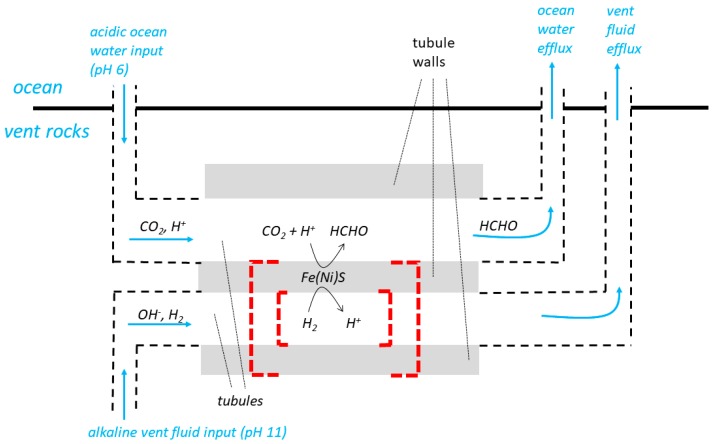
The labyrinthine-network model. From Figure 3 of [18]. The horizontal grey bars represent the inorganic walls of vent-fluid-carrying, and ocean-water-carrying, tubules during the primitive operation of the hypothetical system. The dashed black lines show the source of the alkaline vent fluid and of the more acidic ocean, and the efflux pathways to the ocean. The red dashed lines show how, following the deposition of a lining of the inorganic membrane by organic (lipid) molecules, the tubules seal off to form vesicles following [10].

A third and more recently devised system for the natural pH gradient hypothesis describes how “juxtaposed hydrothermal fluids and ocean waters percolate through a labyrinthine network of interconnected micropores/microcompartments bounded by inorganic membranes” [6,10,14,18,19,29]. To avoid confusion in the terminology, the “interconnected micropores/microcompartments” [6,10,14,18,19,29] are called “tubules” in the following paragraphs and in Figure 3 which is otherwise based on the diagram (Figure 3) from [18]. The diagram includes a representation of a hypothetical, inorganic ΔpH-driven molecular machine catalysing the reduction of CO_2_ to formate and formaldehyde in the inorganic barrier between adjacent tubules. The term molecular machine is used in a broad sense (see Figure 1), and is not intended to misrepresent the CO_2_-reducing device described in [6] (compare [18] and [5,7]). By the definition in Figure 1, and *contra* [18], there *has to be* a ΔpH-driven molecular machine within a functioning framework of the natural pH gradient hypothesis—otherwise no useful chemical work can be performed.

The labyrinthine-network model relies on continuous percolation, flow, in adjacent tubules. Low pH solution is said to have flowed from the ocean into a tubule (e.g., the upper tubule of Figure 3), and high pH solution from the vent into an *adjacent* tubule (e.g., the lower tubule of Figure 3) sharing the same wall. Flow through the tubules from the ocean and the vent would have constantly maintained the ΔpH needed to drive the molecular machines. The exhaust solutions must have been, at least eventually, discharged into the ocean (Figure 3). Central to Lane’s Figure 3 is the implication that a photomicrograph of the modern-day alkali vents at Lost City from [30] supports the model, but this is difficult to accept. Firstly, the Lost City vents are constructed predominantly from Mg(OH)_2_ and CaCO_3_, not metal sulphide. Secondly, there is no suggestion from the photomicrograph or from elsewhere in [30,31] of juxtaposed tubules carrying vent fluid and ocean water.

We now resume our considerations as how the energy supply to ΔpH-driven molecular machines in the membrane tubules of this system might have been managed as the membranes escaped from the vents and became free-living. Again, there are difficulties, but again, we will attempt to follow the arguments of [10]. The inorganic membranes of Figure 3 would have been somehow partially or completely replaced by organic (lipid) membranes and these lipid membranes would then have sealed off to produce vesicles [10]. We show this by the dashed red lines in Figure 3, keeping to the supposition [9], that primitive membrane systems would have had the same polarity of proton pumping as in prokaryotic modern cells. The dilemma for escaping organisms now resurfaces: at the point of sealing off, and without pre-evolved ion pumps, the resulting vesicles would have been released from the vent into the ocean and left without a supply of alkaline fluid to maintain ΔpH to drive ΔpH-dependent molecular machines—the emerging organisms would have perished (see above and [10]). Once again, it is difficult to accept the evolution of H^+^ pumps, SPAP or Na^+^ pumps in the labyrinthine membranes *before* sealing off for reasons similar to those given above—to maintain the natural ΔpH, the aqueous solutions in (upper and lower) tubules on either side of the early membranes *before* sealing off would have been contiguous with the very large volumes of the ocean and the vents (Figure 3). Therefore, ion pumping would not have resulted in significant, useful gradients. Such ion pumps would have been without function before sealing off, and were therefore unlikely to have evolved to assist in the subsequent difficult transition to free-living organisms.

A variant of the labyrinthine-network model, perhaps thought to represent a later evolutionary stage than that in Figure 3, is shown in Figure 1 of [18]. In this variant, the organization of the juxtaposed inorganic vent (alkaline) and ocean (acid) tubules is essentially similar to that shown in Figure 3. However, a linear array of *additional* vesicular compartments, bounded by thinner, organic (lipid) membranes, is arranged or packed within the alkali tubules. Confusingly, the internal pH of these vesicles is shown to be maintained at a high value by alkaline fluid flowing within the tubule and *through* the vesicular lipid membranes. This model raises a number of questions about the relationship with the more primitive model (Figure 3 of [18]), and about fluid flow and H^+^/OH^−^ fluxes through the tubules, across their inorganic walls and across the membranes of the lipid compartments, but plausible explanations for these difficulties are not supplied in the caption of Figure 1 of [18], elsewhere in that publication or in others from the Lane group.

## 5. Escape from the Vents of Lipid Vesicles Trapped in Planar Inorganic Membranes

**Figure 4:**
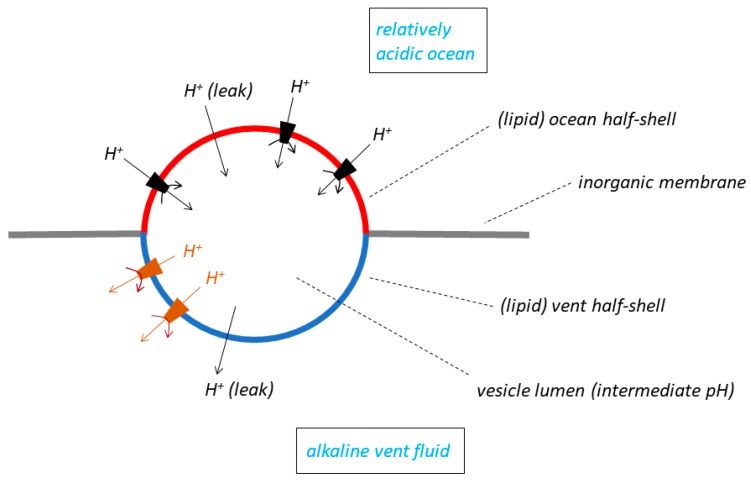
The membrane model serving the mathematical analysis of [32]. The red and blue semi-circles represent the two half-shells of a lipid vesicle trapped in a hole in an inorganic membrane (horizontal grey line) separating the alkaline vent solution from the relatively acidic ocean. The black trapezoids represent inorganic molecular machines capable of utilising the natural ΔpH to drive a useful chemical reaction. The black trapezoid machines are located *only* in the half-shell facing the ocean. The arrows not associated with trapezoids represent proton leaks. Equivalent movements of OH^−^ in the opposite direction are not shown. The brown structures show molecular machines inserted into the vent half-shells in a variant [18] of the earlier model [32]—see text.

We now turn to a fourth model of the membrane systems that have been considered by the RML groups to function in the natural pH gradient hypothesis [32]. This model is said to be set in the context of the LUCA while the organism was still, according to [10], using a natural pH gradient to drive its molecular machines. The model is rather complex (Figure 4): it has a lipid vesicle trapped, or lodged, in a hole in a planar inorganic membrane that separates the alkaline vent fluid from the ocean. The outside of one “half-shell” of the vesicle is exposed to alkaline vent water, and the outside of the other half-shell to relatively acidic ocean water. The insides of both half-shells contact the lumen of the vesicle, which adopts an intermediate pH. The overall pH gradient between vent and ocean thus lies across the *two* half-shells of lipid membrane, that separating the vent from the vesicle lumen, and that separating the lumen from the ocean. The pH drop between the vent and the ocean, usually supposed by the RML groups to be in the region of 3 or 4 pH units (see the compilation in Table 1 of [8]) is thus shared (and therefore decreased) across the membranes of the two lipid half-shells; this is in contrast to the single membrane in either the bleb model (Figure 1A) or the labyrinthine network model (Figure 3), which in principle experiences the full pH drop. The pH gradient has an opposite polarity (inside the vesicle relative to outside) in the two half-shells: acid-to-alkaline across the vent half-shell, and alkaline-to-acid across the ocean half-shell. Sojo et al. [32] insert protein molecular machines capable of utilising the energy of the (shared, decreased) natural ΔpH—they choose an ATP synthase as an example—*only* into the lipid membrane of the ocean half-shell. The machines are *not* inserted into the vent half-shell for reasons of “simplicity” [32] but this may be a little disingenuous. Machines in the vent half-shell with the same orientation as those in the ocean half-shell would be driven *backwards* because of the opposite polarity of ΔpH, which is a much more important reason for avoiding them in this half-shell of the membrane. Lane modifies this arrangement in his Figure 2 of [18] but in a rather startling manner: in the latter publication, he places molecular machines also in the vent half-shell but with an *opposite orientation* to those in the ocean half shell—the former would have produced ATP in the vesicle lumen, the latter in the vent (the ΔpH driving force across each half shell depends on the difficult choice of membrane permeability coefficients).

The model of [32] allows us to resurrect, at least in principle, the mechanisms proposed [10], and rejected above, to explain how the chemiosmotic energy supply might have been managed during escape of the LUCA from the vents. Thus, we could allow the functioning membrane of the LUCA before escape to have been a trapped-vesicle system like that described [32], and we could permit development of outwardly directed protein proton pumps, restricting their location to *just* the ocean half-shell of the lipid vesicle. In contrast to the failure of a proton pump to create a pH gradient between ocean and vent across the single membrane (see earlier section), either of a bleb (Figure 1A) or of a labyrinthine tubule (Figure 3), an emerging pumping activity in the ocean half-shell of the trapped vesicle could have had function: pumping protons into the large-volume ocean would again have had no significant effect on its pH, but the loss of protons from the small-volume, perhaps low-buffering capacity lumen of the vesicle would have resulted in a pH increase in that phase. In this model, before the escape of membrane vesicles from the vents, a pumped ΔpH could indeed have supplemented (to some extent) the natural ΔpH in driving a molecular machine in the vesicle membrane, and possibly have given some survival value to the LUCA. The evolutionarily refined, acquired pump could then have taken over from the natural ΔpH to drive ATP synthesis after dissociation of the trapped vesicle from the vent. If the Na^+^ route of [10] is preferred, we could alternatively consider the development of an SPAP and Na^+^ pump, but again only if their location had been restricted to just the ocean half-shell of the trapped vesicle of [32] before escape. By lowering the Na^+^ concentration in the lumen during pumping (though not significantly changing that in the ocean), a Na^+^ gradient would have been generated across the ocean half-shell, which could have driven a sodium-motive molecular machine before and after escape. Whether we judge one or other of these two escape strategies to be satisfactory depends on our willingness to accept the contrivance of a trapped vesicle with asymmetrically located ion pumps and molecular machines.

Sojo et al. [32] investigate the ionic flux-force relationships (including those arising from an SPAP) in vent membranes that purportedly used the natural ΔpH to drive asymmetrically located molecular machines in their trapped-vesicle model. However, the analysis of Figure 4 is fraught with problems. Sojo et al. [32] explain that they follow the derivations of [33,34], which are based on a model (the squid giant axon) having only a *single* lipid membrane separating just *two* aqueous phases (inside and outside). How the Goldman-Hodgkin-Katz equations can be applied to a model with effectively *three* functional membranes (the two lipid half-shells, and the inorganic membrane), each with very different ion-flux characteristics, and *three* aqueous phases, requires further justification. Though not explicitly stated, the model of [32] subsumes *three* pH gradients and *three* electric potential gradients (between the vent and the lumen, the ocean and the lumen, and the vent and the ocean). Moreover, there are important extra conductance and electrical capacitance elements across the inorganic membrane between the vent and the ocean which should be considered. A de novo derivation is required for proper analysis of the trapped-vesicle model, but we will proceed with one final point.

In the light of their model, Sojo et al. [32] argued that a lifestyle dependent on natural pH gradients *demanded membranes that were extremely leaky to protons*, that there was a *requirement* for leaky membranes, that membranes leaky to protons were *necessary* to harness the energy of a natural pH gradient. Not having been elaborated any of the pre-2014 RML publications (and rather conflicting comments were made in [10,19]), this striking view was re-emphasised as if a new paradigm in more recent papers [6,14,18,29]. However, despite their strongly worded statements, we can see from the minimal bleb model, Figure 1A, that elevated proton permeability is *not* required of the membrane that houses a molecular machine (however, the latter might be defined). Given that Δψ ≈ 0 (see below), and given a sufficient supply of its substrates (and utilisation, or adequate dispersion, of its products), the key factor determining the rate of operation of a molecular machine is the magnitude of the driving force, ΔpH, across the machine, and this would not by any means be *increased* at elevated proton permeabilities.

The conclusion that there is an absolute requirement for proton-leaky membranes in the natural pH gradient hypothesis [32] is, in fact, a consequence of the peculiarities of the trapped-vesicle model, but the conclusion is then extrapolated to a more general case (including the more primitive inorganic membrane systems). It derives from the deduction [32] that the generation of an unfavourable, opposing membrane potential by proton translocation from the ocean to the lumen through molecular machines in the ocean half-shell of a trapped vesicle must be dissipated by proton leak from the lumen to the vent in the vent half-shell. Perhaps the RML groups should not be too troubled by this unfavourable diffusion potential: it could have been very small, especially if the dielectric constant of the membrane matrix, and of the membrane area, and hence of its capacitance, were relatively large (not unlikely in the context of the model). Thus, moderate amounts of charge moving across the membrane would have given rise to only small electric potentials. Possibly, a more plausible method to dissipate the unfavourable but small diffusion potential in the primeval system, which is not model-specific, would have been simply to include a conduction path to ions *other* than protons that were present in the oceans and vents. No other properties would have been required [5]. The device is well known to experimentalists in modern membrane biology and bioenergetics [35,36,37].

## 6. Discussion and Conclusions

The proliferation of models and their variants describing the membranes that might have served to separate alkali vent fluid from the more acidic ocean water in the natural pH gradient hypothesis has become a little confusing. All four of the models outlined above seem to be in current use; they have all been represented in publications from the RML groups within the last five years and are often conflated in discussions of the hypothesis. The RML research groups might consider whether any of the models should be shelved or abandoned or, at least, how ongoing experiments, analyses and observations might lead to their elimination. It may be appreciated that numerous other theories on the origin of life invoke an early involvement of inorganic or lipid membranes: some of the considerations made by the RML groups on the striving for independence of membrane blebs, bubbles and compartments may also apply in a more general sense.

It was suggested [9,10] that developing primitive organisms in vents continued to use the natural ΔpH as an essential energetic intermediate in metabolism until they evolved into the LUCA (on whose chemical definition I have chosen not to comment). The LUCA then escaped from the vents to become a free-living organism in the ocean, but a crucial problem for the RML hypothesis is how the organism managed its energy requirements *during* the escape process. The RML groups propose that escape was achieved by the LUCA membrane bubbling-off or sealing-off from the vent. However, consequent on this process, what remained of the natural ΔpH would have been quickly consumed or dissipated, the organism would have become depleted in the energy supply needed to drive essential molecular machines such as the ATP synthase, and perished. Lane and Martin [10] considered the possibility that ion pumps (either H^+^ or Na^+^) might have developed in the LUCA membrane *before* the sealing-off process began. They argued that the pumps could have generated ion concentration gradients across that membrane to drive molecular machines during and after the subsequent escape, thus providing an immediate replacement for the natural ΔpH. In this article, we make the general case that ion pumps could not have produced ion concentration gradients across the LUCA membrane before sealing off. Thus, the two aqueous phases separated by the LUCA membrane (the vent-fluid system and the ocean) were very large—the uptake of ions from the one and release of ions into the other would not have significantly changed the ion-concentration gradient: there would have been no selective pressure for acquisition and development of the pumps (H^+^ pumps, SPAP or Na^+^ pumps). These arguments apply to bleb membranes [9,12,13,14], to compartments in ocean floor mounds lying on vent seepage sites [1,10,12,17,19,20,21,22,23,24,25,26], and to labyrinthine membrane networks [6,10,14,18,29]. Because of the extra compartment in the trapped-membrane-vesicle model, our arguments do not apply to that system as devised [32]. However, this model is extraordinarily complex with very demanding requirements, and it is doubtful that such a membrane system could have assembled at the origin of life or during the development of the LUCA. It is possible, in fact, that Sojo et al. [32] did not intend their model to be *realistic*, that its properties were chosen either to make the system amenable to their understanding and analysis, or to reveal and amplify concealed but important features. In that case, it should have been incumbent on the authors [32] to discuss which features were included only for those reasons, and which were thought truly to represent the properties of natural membranes in the primitive vents. Presently these arguments are not made, and we must take their description at face value. However, it then becomes difficult to see how the model fits into previous evolutionary schemes of the RML groups, and indeed how such a system could have arisen in any natural environment. The possibility that a population of lipid vesicles (protocells?) suspended in either the ocean or vent fluid became trapped within pre-formed holes of just the right diameter in inorganic membranes separating the ocean and vent (see Figure 4) seems unlikely. The vesicles and their embedded molecular machines could obviously not have utilised the natural ΔpH before entrapment, and the logic is becoming circular. We must add to this the asymmetric location of SPAP, ion pumps and ΔpH-driven molecular machines in *only* one half-shell of the lipid vesicles [32], and the perceived development of the system becomes extremely implausible.

We conclude that realistic versions of the models proposed by the RML groups as functional membranes in the natural pH gradient hypothesis could not have escaped from the alkaline vents. Rather they would have been imprisoned in the vents to await their doom. A heterotrophic origin of life, perhaps in a freshwater environment in hydrothermal fields [38,39,40], may be preferred. Thus, a more likely, if more prosaic, explanation for the evolution of oxidative phosphorylation in ancient organisms is that relatively simple redox-driven proton pumps arose first, and were capable of generating pH gradients to drive uptake of useful weak acids from the environment into the cell [41,42], a process not requiring sophisticated molecular machinery. Primitive forms of the ATP synthase, able to capitalize on the pump-generated ΔpH, arose later [43]. In this context, the power of phylogenetics, and most assuredly of contemporary microbial physiology, to predict events close to the origin of life should not be exaggerated [18]. Moreover, to describe Koch’s opinion [41,42] on the function of a redox pump to drive the uptake of weak acids into primitive cells as “trivial” [18] might be inappropriate. Rather, it may be noted that simple thermodynamics informs us that a modest ΔpH of just two units can drive a >100-fold accumulation of weak acid, of carboxylate for example, and this could have been extremely important in early metabolism.
